# *Smad7* siRNA inhibit expression of extracellular matrix in trabecular meshwork cells treated with TGF-β2

**Published:** 2012-07-11

**Authors:** Ying Su, Chen-Yuan C. Yang, Zhongrui Li, Feng Xu, Lei Zhang, Feng Wang, Shiguang Zhao

**Affiliations:** 1 Department of Ophthalmology, First clinical college of Harbin Medical University, Harbin, China; 2 Department of Anatomy and Neurobiology, Department of Ophthalmology, Boston University School of Medicine, Boston, MA; 3 Department of Neurosurgery, First clinical college of Harbin Medical University, Harbin, China

## Abstract

**Purpose:**

Extracellular matrix (ECM) deposits lead to elevated resistance of aqueous humor outflow which play an important role in the development of primary open angle glaucoma (POAG). The TGF-β2 (transforming growth factor β)/Smad (signaling mathers against decapentaplegic) pathway is known to regulate the ECM deposits. In this study, we determined the effect of *Smad7* siRNA transfection in inhibiting the expression of ECM components.

**Methods:**

Plasmid containing *Smad7* siRNA was used to transfect cultured human trabecular meshwork cells (HTM). Protein expression of Smad7, fibronectin, and laminin was determined using western blot.

**Results:**

Downregulation of *Smad7* interrupts the effects of *TGF-β2* on the expression of several ECM components. *Smad7* siRNA can partially decrease the expression of Smad7, fibronectin, and laminin.

**Conclusions:**

*Smad7* plays an important role in regulating the ECM protein in the aqueous outflow pathway.

## Introduction

Primary open angle glaucoma (POAG) is a leading cause of blindness in the world [1,2]. Elevated intraocular pressure (IOP), consequence of high resistance to aqueous outflow (AH), is an important risk factor in the development and progression of POAG [2]. Hynes [3] has shown that elevated IOP is associated with increased in outflow resistance in the trabecular meshwork (TM) and is related to elevated deposition of extracellular matrix (ECM) material within the TM. Recent studys have found that transforming growth factor-beta 2 (TGF-β2), known to regulate the ECM metabolism including fibronectin, collagen, and elastin, is elevated in the aqueous humor and TM of the glaucoma patient [3,4]. Since the TGF-β/Smad (signaling mathers against decapentaplegic) pathway is important in regulation of ECM deposition in the TM [5], inhibitory Smad7 could potentially antagonize TGF- β/Smad dependent signaling, which induces degration of TGF-β receptor and prevents phosphorylation of Smad2/3 [6,7].

In t5he present study, we determined effect of *Smad7* siRNA in inhibiting the expression of ECM components, including fibronectin and laminin in human trabecular meshwork (HTM) cells.

## Methods

### Trabecular meshwork cell culture and TGF-β2 treatment

Cultures of HTM cells were established from the eyes of five human donors. The research adhered to the tenets of the Declaration of Helsinki. Written informed consent was obtained from all the patients before tissues were collected. This study and all the procedures were approved by the Ethics Committee of the University of Harbin Medical University. The dissection procedure was performed with sterile instruments under a laminar flow hood. The lens, cornea, retina, iris, and ciliary body were extracted first. Then HTM cells between Descement's membrane and the scleral spur were dissected using fine forceps and placed in a 35 mm^2^ culture dish where cells were adhered to the plastic. The cell culture medium, Dulbecco’s Modified Eagle’s Medium (DMEM; low glucose) supplemented with 10% fetal bovine serum (FBS), L-glutamine (0.292 mg/ml), penicillin (100 units/ml), streptomycin (0.1 mg/ml), and amphotericin B (4 mg/ml; HyClone Labs, Logan, UT), was changed every 2 days. HTM cells between passages 5 and 8 were [8-10]. For the TGF-β2 (Sigma Aldrich, St. Louis, MO) treatment group, cells were serum starved for 24 h before treatment with 1 ng/ml TGF-β2 for 24 h [11,12].

### Construction of plasmid with *Smad7* siRNA

Vector pSuppressorNeo (Imgenex, San Diego, CA) is a vector used to generate biologically active siRNAs from the U6promoter. Synthetic oligonucleotide primers (5′- AGG UCA CCA CCA UCC CCA CUU-3′ and 5′-GUG GGG AUG GUG GUG ACC UUU-3′) were annealed and introduced into pSuppressor Neovector [13].

### Transfection HTM with pSup-*Smad7* siRNA

HTM transfected with plasmid containing pSup-*Smad7* siRNA, empty vector only, or medium were served as experimental, vehicle control, and blank control groups, respectively. Transfection was performed in 60 mm plates using 3 µg (1 µg/µl) vector in 10 µl of Metafectene Pro reagent (Biontex, Martinstried, Germany). After 48 h of transfection, cells were treated with G418 (HyClone Labs) for 2 weeks for positive clone selection. After G418 treatment, several stable transfected cells were cloned. Each clone was screened for expression of HTM by western blot analysis [14].

### Western blot analysis

Conditioned medium was collected from HTM cells after treatment with *Smad7* siRNA in serum-free medium containing 0.5 mg/ml BSA (HyClone Labs). Protein concentration was measured using absorbance spectroscopy. Protein was separated on a 10% SDS-polyacrylamide gel and transferred to nitrocellulose membranes. After blocking with 5% nonfat milk, membranes were incubated with primary antibodies against Smad7, fibronectin, and laminin (Santa cruz biotechnology Inc., Santa Cruz, CA) overnight at 4 °C, followed by incubation with secondary antibodies. The membrane was then assayed using the enhanced chemiluminescent kit (ECL, Thermo Scientific, Rockford, IL) and scanned with ChemiDoc™Doc XRS+ system (Bio-Rad, Hercules, CA). The density of each band was obtained using Quantity One 4.6.2 basic software (Bio-Rad). Values were expressed as fold change relative to control and normalized to a loading control, glyceraldehyde 3-phosphate dehydrogenase (GAPDH; Santa Cruz biotechnology Inc.) [15].

### Statistical analysis

The data were analyzed by the two-tailed Student *t*-test using SPSS 10.0 (IBM Inc., Beijing, China) and a p<0.05 was considered significant.

## Results

### Downregulation of Smad7 after transfection with pSup-*Smad7* siRNA

**Figure 1:**
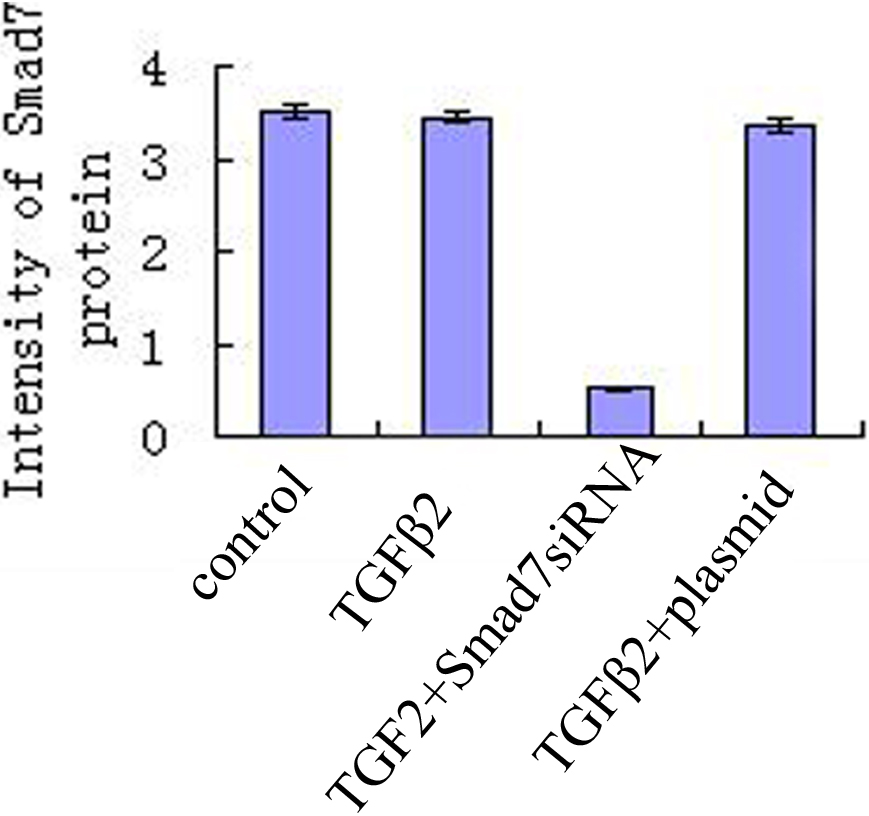
Downregulation of Smad7 after transfection with pSup-*Smad7* siRNA. There was significance among the expression of Smad7 in HTM transfected with Smad7 siRNA, TGF-β2 group, control group, and TGF-β2 plus vehicle group (p<0.01).

Decreased expression of Smad7 was detected in HTM transfected with *Smad7* siRNA compared with the TGF-β2 group, control group and TGF-β2 plus vehicle group (p<0.01; Figure 1).

### Downregulation of expression of fibronectin by transfection with pSup-*Smad7* siRNA

**Figure 2:**
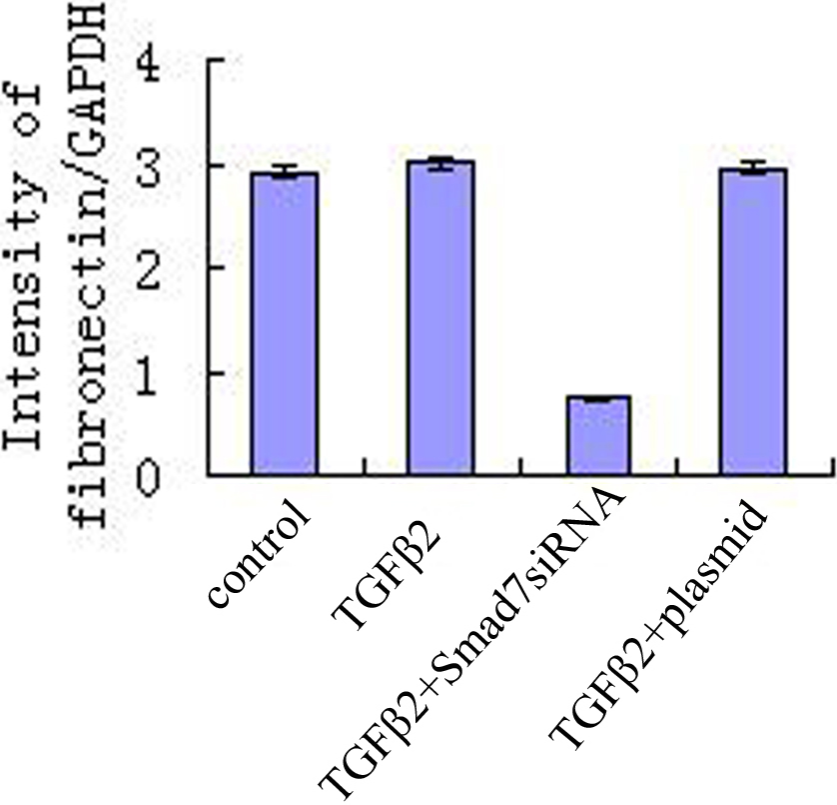
Downregulation expression of fibronectin by transfection with pSup-*Smad7* siRNA. Fibronectin is a glycoprotein of the extracellular matrix that binds to membrane-spanning receptor proteins called integrins. There is significance among expression of fibronectin protein in the HTM transfected with Smad7siRNA, TGF-β2 group, control group, and TGF-β2 plus vehicle group (p<0.01). Downregulation of fibronectin was detected in HTM transfected with pSup-*Smad7* siRNA.

Fibronectin protein was expressed in the HTM transfected with *Smad7* siRNA, TGF-β2 group, control group and TGF-β2 plus vehicle group (p<0.01). Downregulation of fibronectin was detected in HTM transfected with pSup-*Smad7* siRNA (Figure 2).

### Inhibition of expression of laminin after transfection with pSup-*Smad7* siRNA

**Figure 3:**
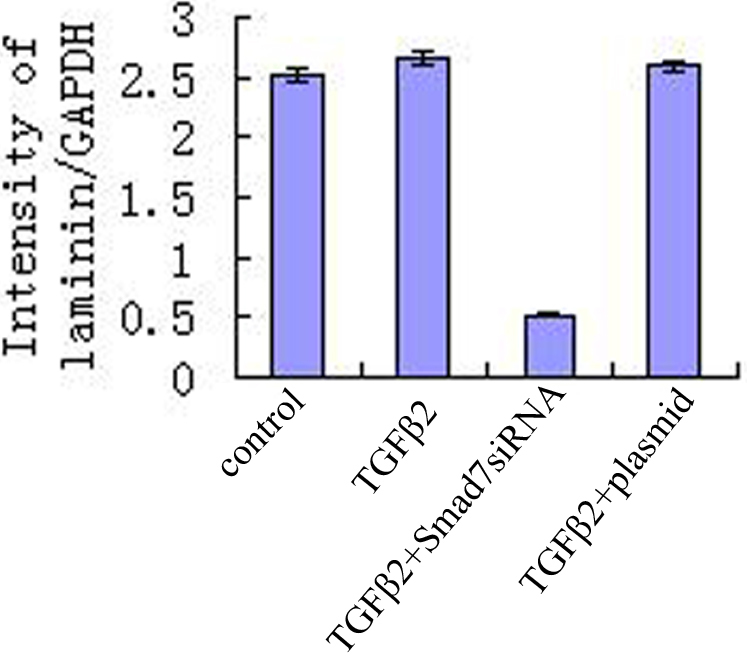
Inhibit expression of laminin after transfection with pSup-*Smad7* siRNA. There is significance among expression of laminin protein in the HTM of Smad7siRNA group, TGF-β2 group, control group, and TGF-β2 plus vehicle group (p<0.01). Downregulation of laminin was detected in HTM transfected with pSup-*Smad7* siRNA.

Laminin protein was expressed in the HTM transfected with *Smad7* siRNA, TGF-β2 group, control group, and TGF-β2 plus vehicle group (p<0.01). Downregulation of laminin was detected in HTM transfected with pSup-*Smad7* siRNA (Figure 3).

## Discussion

Abnormal accumulation of ECM components can cause fibrosis [16-18]. Additionally, irregularities in multiple pathways involved in tissue repair and inflammation can lead to the development of heart, kidney, lung fibrosis [19,20]. ECM components such as collagens, fibronectin, laminin, and elastin affect the development of fibrosis [21-23]. Similarly, abnormal accumulation of ECM can lead to elevated resistance in the TM, especially in the juxtacanalicular region, which has been shown to be the major resistance site for the elevated IOP of POAG in the aqueous outflow pathway [24,25].

TGF-β2 is an important ligand that modulates cell behavior in ocular tissues including enhancing ECM production [26,27], and its gene targets are activated by the translocation of Smad2 and Smad3, and their common mediator Smad4 [28,29]. There are three groups of Smad proteins: receptor-regulated Smads, common-partner Smad, and inhibitory Smads [30-32]. Inhibition of TGF-β family signaling by Smad6 and Smad7 through multiple mechanisms plays a critical role in various physiologic processes [33]. Decreased expression of Smad7 has been reported in patients with scleroderma and inflammatory bowel disease [34].

Our study showed that transfection with pSup-*Smad7* siRNA can downregulate Smad7 expression of cultured HTM cells. In summary, this study has delineated the role of Smad7 downregulation in inhibition the expression of ECM protein including fibronectin and laminin of cultured HTM. Downregulation expression of fibronectin and laminin after transfection with pSup-*Smad7* siRNA suggest its role in the TGF-β2/Smad pathway in regulating ECM deposits.

In conclusion, the present work demonstrates for the first time that transfection with pSup-*Smad7* siRNA can effectively inhibit ECM deposits of HTM treated with TGF-β2 in vitro which indicates it can be novel target for treatment of POAG. Although Smad7 is a powerful way to inhibit the expression of ECM components, further work is needed before this approach can be used for the treatment of human disease.
